# The origin of T-tubules

**DOI:** 10.7554/eLife.88954

**Published:** 2023-06-20

**Authors:** Callum J Quinn, Katharine M Dibb

**Affiliations:** 1 Unit of Cardiac Physiology, Manchester Academic Health Science Centre, University of Manchester Manchester United Kingdom

**Keywords:** caveolae, T-tubules, caveolin 3, correlative microscopy, caveolinopathie, skeletal muscle, Human, Mouse

## Abstract

Ring-like structures made up of caveolae appear to drive the development of membrane invaginations called T-tubules which are important for muscle contraction.

**Related research article** Lemerle E, Lainé J, Benoist M, Moulay G, Bigot A, Labasse C, Madelaine A, Canette A, Aubin P, Vallat J-M, Romero NB, Bitoun M, Mouly V, Marty I, Cadot B, Picas L, Vassilopoulos S. 2023. Caveolae and Bin1 form ring-shaped platforms for T-tubule initiation. *eLife* **12**:e84139. doi: 10.7554/eLife.84139.

In order to move, skeletal muscles connected to the bones of the body must be triggered to contract. This requires nerves in the brain to activate cells within muscle to release calcium ions from their internal store, the sarcoplasmic reticulum. Electrical signals sent from the brain are transmitted to the centre of the muscle cell by invaginations in their plasma membrane, called T-tubules, which are closely associated with the sarcoplasmic reticulum (Franzini-Armstrong, 2018; Smith et al., 2018). This results in the synchronous release of calcium ions throughout the whole cell, ensuring efficient contraction of the muscle.

Mutations in genes that coordinate the development of T-tubules have been associated with debilitating muscle diseases (Fujise et al., 2022). While some of the proteins involved in this process have been identified, such as the membrane shaping protein Bin1, we do not fully understand how T-tubules mature and grow alongside the sarcoplasmic reticulum. Early studies suggest that cave-like pits within the plasma membrane called caveolae – which are found in multiple cell types, including muscle cells – may play a role (Parton and Simons, 2007; Franzini-Armstrong, 1991). However, a lack of appropriate tools has made it difficult for researchers to visualise how this complex process occurs. Now, in eLife, Stéphane Vassilopoulos from Sorbonne Université and colleagues – including Eline Lemerle as first author – report that T-tubules grow from sub-membrane rings composed of caveolae and Bin1 in mammalian skeletal muscle cells (Lemerle et al., 2023).

The team (who are based at multiple institutes in France) studied aggregates of developing human or mouse muscle cells grown in the laboratory, known as myotubes. Using ultrasound, Lemerle et al. removed the upper cell membrane of the myotubes so that the inner side of the plasma membrane could be observed. This revealed rings of plasma membrane closely associated with the sarcoplasmic reticulum, which were coated in the caveolae-forming protein Cav3. A state-of-the-art technique that combines light and electron microscopy (known as CLEM) showed that some of these constructs – which Lemerle et al. termed caveolae rings – had multiple tube-like structures extending from them. Present on the tubular extensions were Bin1 proteins and calcium channels, two components associated with T-tubules, suggesting that these structure may be precursors to T-tubules.

It has been suggested that Bin1 induces membrane curvature and promotes T-tubule development (Lee et al., 2002). By modifying developing muscle cells to contain more of a type of Bin1 protein with a specific lipid-binding domain, Lemerle et al. increased the formation of circular structures – resembling caveolae rings – from which T-tubules could sprout. While all the structures were coated in Bin1, only some were associated with Cav3. Furthermore, when high concentrations of this Bin1 protein were added to a semi-synthetic membrane, the membrane self-assembled into rings and T-tubules similar to those that naturally develop in lab-grown cells. These findings suggest that Bin1 is primarily responsible for organizing the plasma membrane into ring-like platforms which then recruit caveolae and sprout T-tubules (Figure 1).

**Figure 1.**
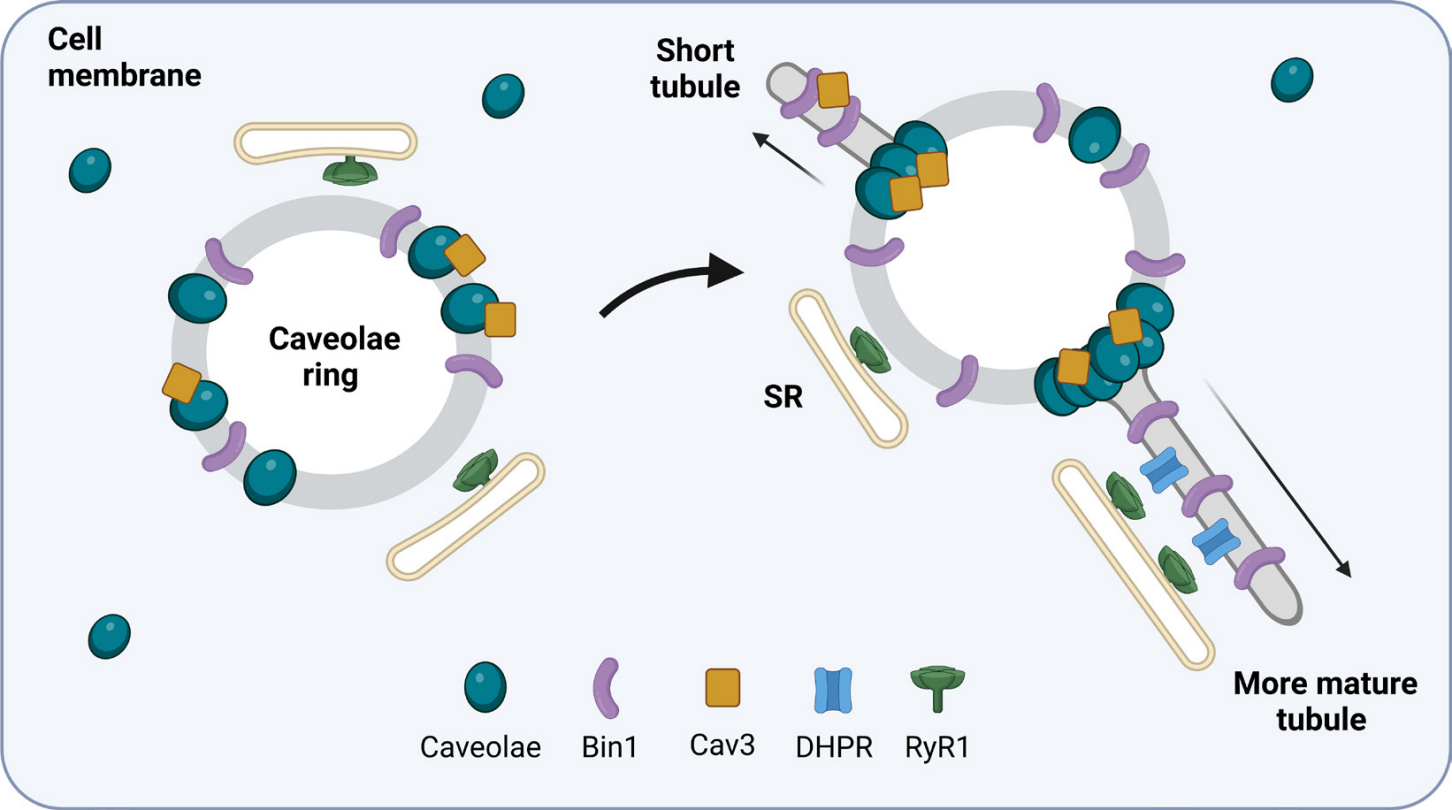
How T-tubules form in developing muscle cells. The protein Bin1 (purple) reshapes the plasma membrane to form rings that sit beneath the membrane of the skeletal muscle cell. Cave-like pits in the plasma membrane called caveolae (blue) are then recruited to the ring together with the caveolae-forming protein Cav3 (yellow). This results in structures termed caveolae rings which associate with the sarcoplasmic reticulum (SR) at the sites where caveolae are not present. Cav3 proteins and caveolae then accumulate at specific locations on the ring and tube-like structures start to emerge from these sites. As the tubule matures, calcium channels called dihydropyridine receptors (DHPRs; blue) begin to form a calcium release unit with ryanodine receptor 1 (RyR1; green) on the membrane of the sarcoplasmic reticulum. This interaction resembles how T-tubules and the sarcoplasmic reticulum associate with one another in mature skeletal muscle cells.

Next, Lemerle et al. reduced the amount of Cav3 expressed in the myotubes to better understand the role caveolae play in T-tubule formation. This resulted in smaller rings and a dramatic decrease in tubulation, suggesting that caveolae are important for this process. The team then analysed developing muscle cells that had been genetically modified to contain mutations in the gene for Cav3 which are known to cause human muscle diseases called caveolinopathies (Woodman et al., 2004). As expected, this resulted in aberrations in how caveolae were organized and disrupted tubulation in the mutant muscle cells, which correlated with the mature T-tubule malformations observed in tissue extracts from patients with caveolinopathies.

In this breakthrough study, Lemerle et al. have presented a compelling new mechanism for how T-tubules develop. In addition to making a significant contribution to skeletal muscle research, these findings likely relate to cardiac muscle cells which also require Bin1 and Cav3, amongst other proteins, to form T-tubules (Caldwell et al., 2014; Bryant et al., 2018). Faults in cardiac T-tubules have been associated with heart failure, arrhythmias, and other cardiac diseases (Smith et al., 2018; Smith et al., 2022). Therefore, the findings and experimental approach used by Lemerle et al. could help researchers better understand how T-tubules can be targeted to treat muscle diseases that impact the heart as well as those associated with movement.
